# Paediatric Neurology: Current Trends, Rehabilitation, and Future Challenges

**DOI:** 10.3390/jpm14010077

**Published:** 2024-01-09

**Authors:** Domenico M. Romeo, Claudia Brogna

**Affiliations:** 1 Pediatric Neurology Unit, Fondazione Policlinico Universitario A. Gemelli, IRCCS, 00168 Rome, Italy; claudiabrogna@yahoo.it; 2 Pediatric Neurology Unit, Universittà Cattolica Del Sacro Cuore, 00168 Rome, Italy

Over the past 20 years, the introduction of new neurodevelopmental assessments and neurophysiological techniques has improved the knowledge of the complexity of the central nervous system in the first period of development. More recently, different studies in infants at risk of neurodevelopmental impairments reported on novel data about the maturation of specific features of its function and their relationship with neurological tools, helping clinicians in early treatment and rehabilitation programmes.

Children with cerebral palsy, neuromuscular and metabolic disorders, epilepsy, and preterm children represent the main patients in paediatric neurology in terms of frequency, as well as social and economic impact. This Special Issue, containing five articles, focuses on the current evidence of evaluation tools, new technologies, and intervention approaches for paediatric patients with these neurological impairments.

Clinical and electrophysiological evaluations represent the first level of assessment in paediatric neurology. Preterm infants (mainly those born at a gestational age < 32 weeks) report lower scores on neurodevelopmental tests and are at greatest risk of developmental problems compared to those born at term. In the paper published by Makila et al. [1], the parental questionnaire Five-to-Fifteen (FTF) was used to explore the parental perception of the developmental profile of children born very preterm from ages 5 to 8 years; very preterm children showed lower scores than peers born at term in most of the tasks explored, especially in gross motor skills, executive function, and language. The FTF should be used as screening tool to early identify preterm children at risk of neurodevelopmental problems and who should be actively referred to a rehabilitation programme. Along the same line of evidence, the review of Romeo et al. [2] explored another clinical instrument, the 6 min walk test (6MWT), as a reliable tool to assess the effect of treatment on the walking ability in children with cerebral palsy (CP). The 6MWT was considered to be an effective instrument to assess the changes in walking abilities in children with different types of CP from 5 years old onwards. The authors concluded that the systematic use of the 6MWT should be proposed in clinical and research settings to promote rehabilitation activities that could maintain or improve walking and/or gross motor function.

Epilepsy is considered one of the most recurrent paediatric neurological conditions. The use of an electroencephalogram (EEG) represents an essential instrument for diagnostic information, helping clinicians to make a decision in terms of pharmacological treatment, especially in children with non-convulsive status epilepticus (NCSE). However, standard EEG recordings are time- and staff-consuming, and their accessibility is restricted, especially outside regular working hours. In their technical note, Simma et al. [3] reported on the application of a simplified EEG recording technique, using a reduced lead montage (point-of-care EEG—pocEEG), for identifying NCSE and managing its treatment. This new instrument allows neuromonitoring of paediatric patients with neurological conditions, simplifying opportune diagnosis and treatments when a standard EEG is not readily accessible.

Neuromuscular and neurometabolic disorders are important group of diseases, mostly presenting in newborns and infants, due to defects or mutations in a single gene. Most of them respond favourably to treatment, especially when started at the beginning of the symptoms. Therefore, early detection and early intervention are considered invaluable in these patients. A contribution to this research topic was reported in two case reports by De rose et al. [4] and Faccioli et al. [5]. In the first one, the authors pointed out the importance of specific early clinical signs, such as bilateral vocal cord paralysis (requiring a tracheostomy) and feeding problems that could be the early diagnostic indications of a congenital myasthenic syndrome (CMS) with onset during the neonatal period due to a mutation in the MUSK gene. These newborns should be referred to III-level centres for neurophysiology and genetic assessments as soon as possible, to avoid a late diagnosis of CMS and improve outcomes. Faccioli et al. [5] described the long-term (5 years) management of the residual walking impairment of a child with late infantile metachromatic leukodystrophy treated with hematopoietic stem cell gene therapy. This therapy in addition to a specific rehabilitation programme, including orthoses, a walker, orthopaedic surgery, physiotherapy, and botulinum toxin preserved survival and locomotor abilities. This multidisciplinary approach is required to preserve long-term gait competence, reduce the incidence of deformities and pain, and guarantee independence in daily life in these patients.

In conclusion, the present scientific studies give us evidence on the progress in Paediatric Neurology and its future challenges. The papers gather contributions from diverse experts in the field of neonatal and paediatric neurology and address specific problems, such as novel diagnostic and screening tools and rehabilitation approaches, adding new understandings and views in the treatment of these disorders. We hope that the information collected from this Special Issue will promote and offer suggestions for future research in this field to better prevent and treat neurological disorders.
